# Unraveling the Morphology of [C_*n*_C_1_Im]Cl Ionic Liquids Combining Cluster and Aggregation Analyses

**DOI:** 10.1021/acs.jpcb.3c08317

**Published:** 2024-04-15

**Authors:** Tom Frömbgen, José Nuno Canongia Lopes, Barbara Kirchner, Karina Shimizu

**Affiliations:** † Mulliken Center for Theoretical Chemistry, University of Bonn, Beringstraße 4-6, D-53115 Bonn, Germany; ‡ Max-Planck-Institut für Chemische Energiekonversion, Stiftstrasse 34-36, D-45470 Mülheim an der Ruhr, Germany; ¶ Centro de Química Estrutural, Institute of Molecular Sciences, Instituto Superior Técnico, Universidade de Lisboa, Av Rovisco Pais 1, 1049 001 Lisboa, Portugal

## Abstract

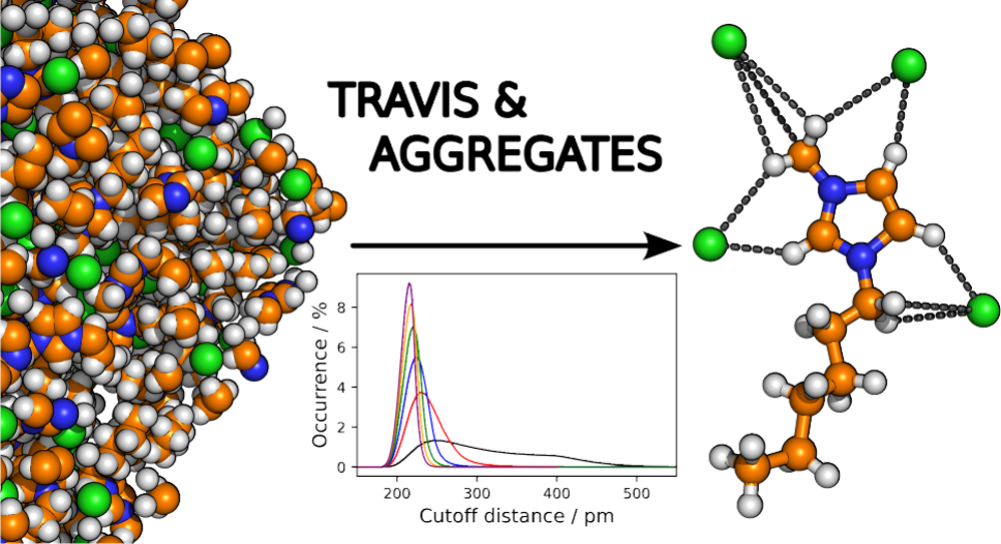

A characteristic feature of ionic liquids is their nanosegregation, resulting in the formation of polar and nonpolar domains. The influence of increasing the alkyl side chain on the morphology of ionic liquids has been the subject of many studies. Typically, the polar network (charged part of the cation and anion) constitutes a continuous subphase that partially breaks to allow the formation of a nonpolar domain with the increase of the alkyl chain. As the nonpolar network expands, the number of tails per aggregate increases until the ionic liquid percolates. In this work, we demonstrate how the complementary software packages TRAVIS and AGGREGATES can be employed in conjunction to gain insights into the size and morphology of the [C_*n*_C_1_Im]Cl family, with *n* ∈ {2, 4, 6, 8, 10, 12}. The combination of the two approaches rounds off the picture of the intricate arrangement and structural features of the alkyl chains.

## 1 Introduction

Ionic liquids (ILs) constitute a well-established field of research, for example, due to their fascinating physicochemical properties that distinguish them from conventional molecular liquids.^1−5^ A particularly interesting facet of ILs is their mesoscopic order, also known as nanosegregation, that has been investigated experimentally and computationally in various studies.^6−9^ Notably, ILs often exhibit a distinct and continuous polar domain comprising the charged (and thus polar) moieties of their constituents. Conversely, the nonpolar moieties tend to form large nonpolar regimes the size of which are dependent on the nature of the IL ions.^10−12^ The formation of polar and nonpolar domains is primarily driven by favorable electrostatic and noncovalent interactions, respectively, and may be hindered by steric demands of the ions. This mesoscopic order can be controlled by the choice of IL constituents, for example, upon elongation of the alkyl side chains of the IL.

In the present work, we focus on the common *n*-alkyl-methyl-imidazolium chloride ILs (with *n* ∈ {2, 4, 6, 8, 10, 12}) and aim at unraveling the morphology of this specific IL family. We acknowledge previous works that investigate the structure and/or related properties of different imidazolium-based ILs or IL families.^13−27^ To commence this investigation, classical nonpolarizable molecular dynamics (MD) simulations of the ILs were performed. Subsequently, cluster and aggregation analyses from TRAVIS^28−30^ and AGGREGATES^31^ were carried out. Both software packages offer a variety of analyses to study the structure of liquids, which can complement each other. We demonstrate how these two complementary software tools synergistically contribute to providing a comprehensive understanding of the structural arrangement of the ILs, particularly in relation to the side chain length of the cation.

## 2 Methods

### 2.1 Systems Investigated

**Figure 1:**
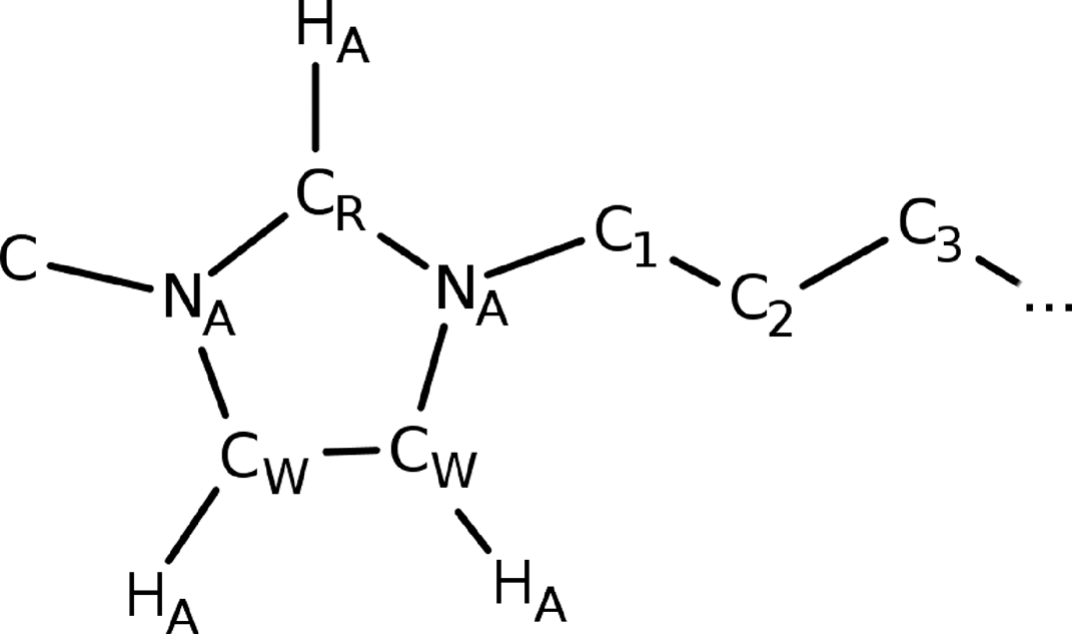
Illustrative Lewis structure of a [C_*n*_C_1_Im]^+^ cation with important atoms labeled.

In this study, we investigate six alkyl-imidazolium-based ionic liquids [C_*n*_C_1_Im]Cl with *n* ∈ {2, 4, 6, 8, 10, 12} by means of classical molecular dynamics simulations. We note that at the simulation temperature of 400 K, all the ILs studied will reside in the liquid state.^32−38^ Phase transitions occur at time scales that are difficult to directly sample using all-atom MD simulations. Klajmon and Cervinka^39^ recently published a study on predicting glass transition temperatures of imidazolium-based ionic liquids, analyzing volumetric and transport properties using different force field approaches. The authors did not observe qualitative or quantitative differences between the radial distribution functions calculated for the glass and liquid phases. Each system comprises 700 ion pairs (IPs). An illustrative representation of [C_*n*_C_1_Im]^+^ with labeling of important atoms that will be addressed in this manuscript is shown in Figure 1. Further computational details are provided in section 2.2.

### 2.2 Computational Details

Molecular dynamics simulations were conducted using DL_POLY 2.20 and GROMACS 2020 packages.^40−45^ In these simulations, we focused on the [C_*n*_C_1_Im]Cl ionic liquid family, where *n* ∈ {2, 4, 6, 8, 10, 12}. The OPLS/AMBER-like CL&P force field was employed to model these ionic liquids.^46−51^ To initiate the simulations, low-density configurations were created, each consisting of 700 ion pairs. These initial configurations were generated using fftool and Packmol software.^52,53^ The simulations were executed with a time step of 2 fs and a cutoff distance of 1200 pm. Subsequently, a simulated annealing process was applied over a 10 ns duration. During the annealing, the temperature was gradually increased from 400 to 550 K. We used a V-rescale thermostat and Berendsen barostat with relaxation times of 0.5 and 4.0 ps, respectively, to control the temperature and pressure. Finally, the system was brought back to 400 K and 1 atm. After annealing, the simulations were further equilibrated under *NpT* ensemble conditions, where the pressure was set to 1 atm and the temperature to 400 K. The V-rescale thermostat and Berendsen barostat were employed again, with relaxation time constants of 0.5 and 2.0 ps. This equilibration phase lasted for 25 ns. The systems achieved a stable and consistent density after 15 ns of equilibration, signifying that equilibrium had been reached and any potential ergodicity issues had been resolved. Subsequently, a 10 ns production stage was conducted using a time step of 1 fs under *NpT* ensemble conditions with 400 K and 1 atm. During the production run, the trajectory was dumped with a frequency of 2000 steps, finally resulting in 5000 trajectory frames with a temporal difference of 2 ps. At this stage, the Nosé–Hoover thermostat and Parrinello–Rahman barostat were utilized with relaxation times of 0.5 and 4.0 ps. The simulation boxes exhibited varying final volumes, ranging from 5400 × 5400 × 5400 pm^3^ to 7200 × 7200 × 7200 pm^3^. To analyze the simulation results, pair correlation functions (*g*(*r*)), cluster analyses, and aggregation analyses were computed using previously established formulas and methodologies as implemented in the TRAVIS and AGGREGATES software.^28−31^ Each analysis was performed using the entire trajectory of 5000 frames.

## 3 Results and Discussion

In this section, the results of different structural analyses of the [C_*n*_C_1_Im]Cl family are presented. First, we discuss the radial distribution functions of cations and anions as well as their contact neighbor analysis in section 3.1. Then, we turn to analyzing the polar and nonpolar domains in section 3.2. Finally, in section 3.3, we discuss the structural arrangement of the alkyl chains with respect to each other and draw conclusions about the IL morphology. Our analyses are consolidated by further material displayed in the Supporting Information (SI).

### 3.1 Radial Distribution and Contact Neighbors

**Figure 2:**
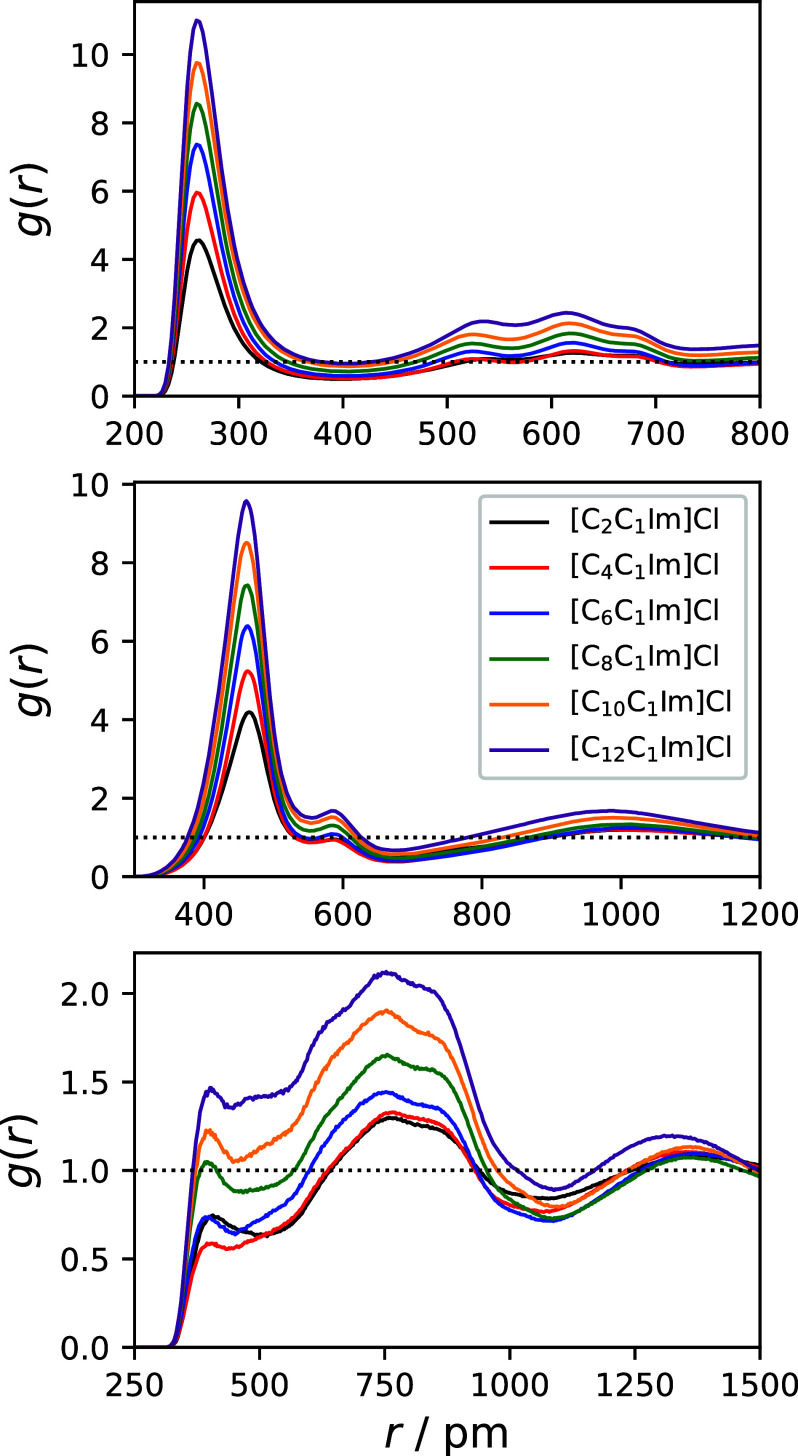
Radial distribution functions: Top panel: Between Cl^–^ and the acidic protons of the imidazolium ring (H_A_, cf. Figure 1 for the notation). Middle panel: Between Cl^–^ and the cation center of ring COR[C_*n*_C_1_Im]^+^. Bottom panel: Between COR[C_*n*_C_1_Im]^+^ and COR[C_*n*_C_1_Im]^+^.

**Figure 3:**
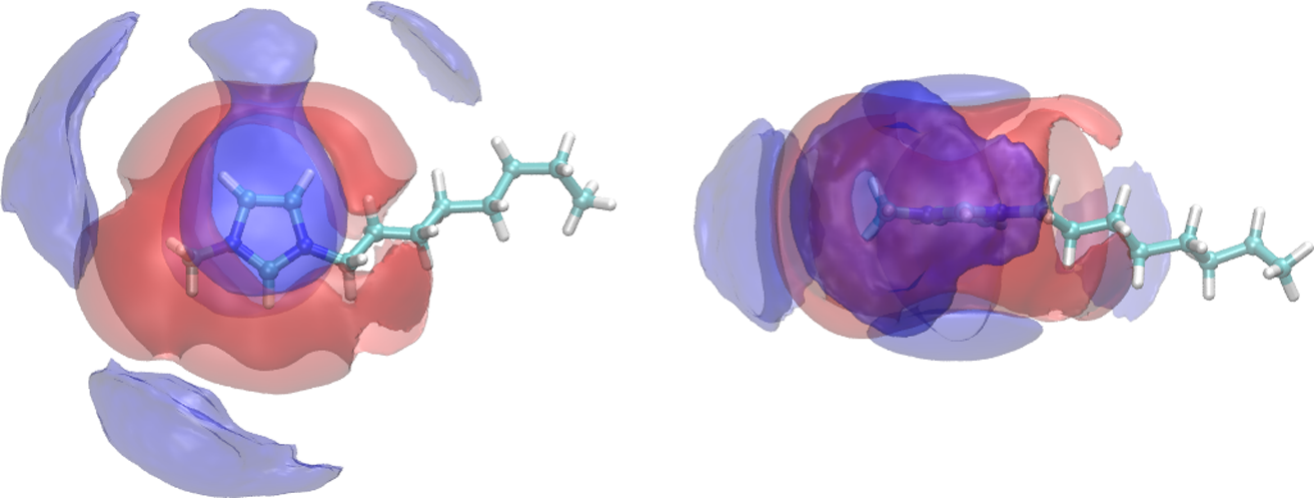
Selected spatial distribution functions around the imidazolium cation of [C_8_C_1_Im]Cl: front view (left) and side view (right). The red and blue colors represent the anion and the cation ring, respectively.

Figure 2 presents radial distribution functions (RDFs) between different components of the studied systems, while Table 1 provides important quantities from these RDFs. The top panels display the hydrogen bonding interaction between Cl^–^ and the imidazolium ring protons H_A_ (cf. Figure 1). Overall, the radial distribution is mostly unaffected by an increased alkyl chain length. The first maxima and minima of the RDFs are observed at around 260 and 400 pm, respectively, with a coordination number of 3.7 to 3.9 in the first solvation shell. It is noteworthy that the absolute *g*(*r*) value rises from *n* = 2 → 12, which is expected when the total number of atoms in the system increases while the number of Cl^–^ and H_A_ atoms remains constant. Notably, there are other important H–Cl^–^ interactions, namely between chloride and the aliphatic CH_2_ and CH_3_ groups attached to the imidazolium ring. Such interactions were already described in ref (54). The corresponding RDFs feature a first peak at 290 pm as well as a reduced peak height compared to that of the H_A_–Cl^–^ RDFs. To see the corresponding figure, we refer the interested reader to Section 1 of the Supporting Information. The spatial arrangement of the cation rings and anions around each other is best visualized in Figure 3. The figure also highlights the aforementioned important interactions of chloride with the aliphatic residues attached to the imidazolium ring.

**Table 1:** Important Quantities from the RDFs Presented in Figure 2^a^

RDF	*n*	*r*_max_	*r*_min_	NI(*r*_min_)
Cl^–^–H_A_	2	262	400	3.9
	4	260	410	3.8
	6	260	398	3.7
	8	260	398	3.7
	10	260	400	3.8
	12	260	406	3.8
Cl^–^–COR	2	465	553	4.4
	4	463	557	4.1
	6	463	553	4.0
	8	463	553	4.0
	10	461	555	4.1
	12	461	555	4.1
COR– COR	2	407	515	1.1
	4	403	433	0.3
	6	399	451	0.4
	8	395	467	0.6
	10	401	447	0.5
	12	403	447	0.5

a Position of the first maximum and minimum, *r*_max_ and *r*_min_, respectively (all in pm), and the coordination number at the position of the first minimum NI(*r*_min_). The three data sets have the same order as the corresponding RDFs.

The center panel of Figure 2 and Table 1 address the radial distribution of the cation’s center of ring (COR) around the anion. Similar to the hydrogen bonding interactions, minimal variations are observed across different systems. The first maxima and minima of the RDFs are consistently located at around 463 and 555 pm, respectively, and the coordination number at the first minimum is found to be around 4.1. Moving to the bottom panels, these show the RDFs between the COR of the cations. Therein, curves do not exhibit a well-defined peak shape as compared to the previous RDFs but feature a small first peak at round 403 pm, followed by a minimum between 433 and 467 pm. It should be noted that the height of the first peak is rather low for certain systems (*g*(*r*) < 1 ∀ *n* ≤ 6). Below *r* = 500 pm, the curves do not follow an exactly identical shape, hampering a fair comparison of their coordination numbers in the first solvation shell, which range from 0.3 to 0.6 without a discernible trend. The system with *n* = 2 represents an outlier inasmuch as its RDF minimum is found at 515 pm, resulting in a coordination number of 1.1. At larger distances, the RDFs align again and show a pronounced second maximum at 760 pm.

In summary, the aforementioned observations are in agreement with previous studies^54,55^ and give rise to the suggestion that, on average, every Cl^–^ is surrounded by four cations and vice versa. Each ion interacts with the other species by one hydrogen bond (mostly), independent of the alkyl chain length. The small initial peak of the COR–COR RDFs could potentially originate from the bulky and rigid nature of the cation ring motif preventing a uniform cation arrangement at short distances and resulting in two preferred distances between cations. However, the arrangement of the cations with respect to each other is not fully clear at this point and will be discussed in the following.

**Figure 4:**
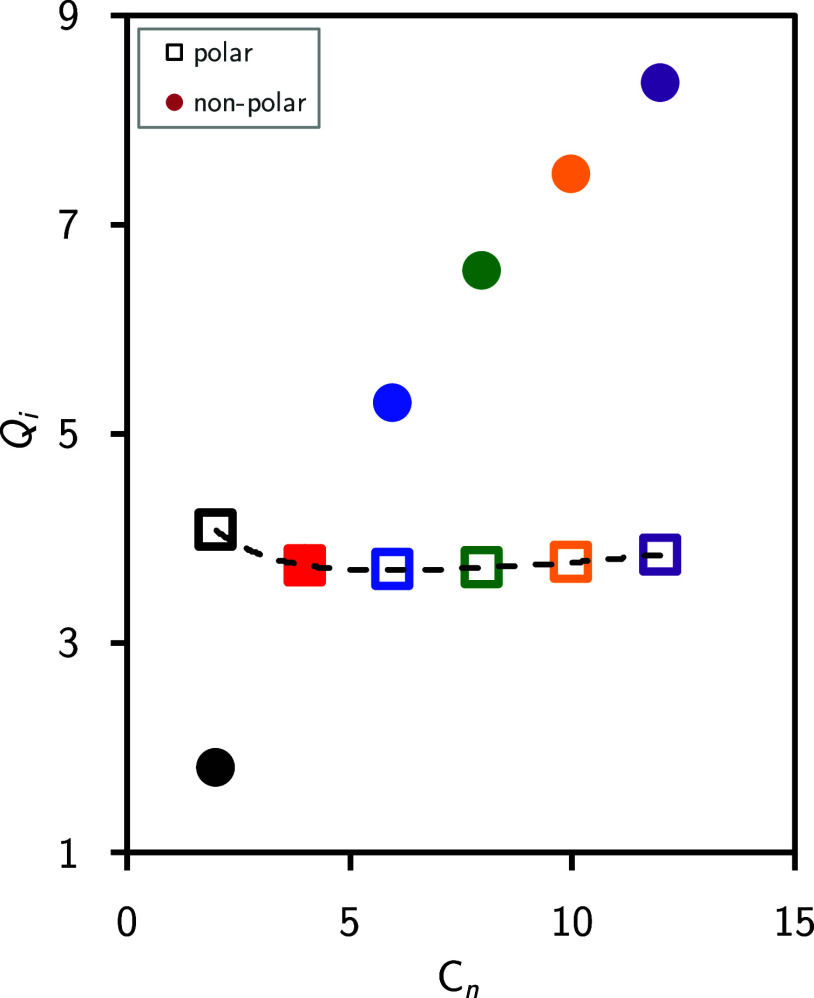
Average size of contact neighbors, *Q*_*i*_, in the polar domain (empty squares) or in the tail aggregates (filled color circles) as a function of alkyl side chain length *n*.

Additional analyses, complementing the radial distribution functions, are presented in Figure 4, focusing on the average size of contact neighbors *Q*_*i*_ in both polar and nonpolar domains as a function of the alkyl side chain length *n*. The number of contact neighbors can be calculated either as a function of the aggregate size containing the polar domain or the alkyl chain, denoted as *Q*_*i*_(*n*_*a*_), or as an average number across the entire simulation box, denoted as *Q*_*i*_ (the latter being presented in Figure 4). It should be noted that the polar domains include the anions as well as the [C_*n*_C_1_Im]^+^ fragments of the cations, while the nonpolar domain comprises the cations tails, starting from the second carbon atom. To be precise, that is *n* – 1 CH_2/3_ groups of each [C_*n*_C_1_Im]^+^ cation tail. This value is associated with the coordination number obtained from the radial distribution functions. In accordance with our aforementioned findings from Table 1, the average number of contact neighbors in the polar domain varies only slightly with increasing *n* and is found to be between 3.7 and 4.0. Intriguingly, in the nonpolar regime, we find a systematic increase of the contact neighbor size when the cation tails are prolonged, namely, from 1.8 to 8.4 for *n* = 2 and *n* = 12, respectively. This trend lets us conclude that the polar network within the ILs is indeed very stable and largely unaffected by altering the cation side chains, while there are significant changes and a growing organization in the nonpolar domains. Further investigations into this evolution are the focus of subsequent analyses.

### 3.2 Domain Analysis

In order to further elucidate the structure of the differently sized ILs and the cation arrangement in particular, domain analyses^56^ were performed. A domain analysis is based on radical Voronoi tessellation of the simulation box. In such an analysis, the entire box is partitioned into geometric polyhedra (Voronoi cells), each of which contains exactly one atom. By sorting atom types into certain groups and subsequently merging the Voronoi cells of atoms from the same groups that share cell faces, significant information about the formation of domains in the systems can be gained. For the domain analyses, four distinct investigations were carried out, each of which included all of the atoms in the base population. The population of the first and second domains comprised the anions and [C_1_C_1_Im]^+^ cation head groups, exclusively, while the third and fourth domain were defined in the previous section. In Table 2, the average domain counts  (anions),  (polar [C_1_C_1_Im]^+^ fragments), *N*_polar_ (anions and polar [C_1_C_1_Im]^+^ fragments), as well as *N*_alkyl_ (nonpolar alkyl chains) are displayed.

**Table 2:** Results from the Domain Analysis^a^

*n*	NCl^–^	N[C_1_C_1_Im]^+^	*N*_polar_	*N*_alkyl_
2	675	1.0	1.0	59.1
4	678	1.0	1.0	3.0
6	679	1.0	1.0	1.6
8	679	1.0	1.0	1.3
10	677	1.0	1.0	1.0
12	678	1.0	1.0	1.0

a All atoms were included in the base population.  denotes the average domain count for a domain population containing all Cl^–^.  denotes the average count of cation head group domains. *N*_polar_ denotes the average domain count for a domain population containing all [C_1_C_1_Im]^+^ groups and all Cl^–^. *N*_alkyl_ denotes the average domain count for a domain population comprising everything not included in *N*_polar_, that is the cations’ alkyl chains, starting from the second carbon atom.

The domain analysis for the anions reveals a consistently large and almost constant domain count ranging from 675 to 679. Given that each system contains 700 anions, this suggests minimal direct Cl^–^–Cl^–^ contacts. Specifically, chloride ions do not form a network but predominantly exist in isolated states, with a few exceptions forming domains of two or three ions. Combining the anions with the cations’ polar fragments in the polar domain analysis results in a constant average domain count of 1.0 for all investigated systems. These observations support the findings from section 3.1 that indicated a pronounced hydrogen bonding network, resulting in a well-defined coordination of the anions by approximately four cations. Intriguingly, the polar cation head groups alone exhibit a constant domain count of 1.0 across all investigated systems. While this behavior might, for systems including short cation chains, originate from the fact that the cation headgroup constitutes the majority of the atoms in the system, the situation becomes different when the chains are prolonged. Even in the systems with very long side chains and without incorporation of the anions, a domain count of unity is observed for the [C_1_C_1_Im]^+^ domain. These observations give rise to a pronounced cation–cation interaction. In contrast, the fourth domain analysis, considering the nonpolar residues (i.e., the cation side chains), demonstrates a steeply decreasing average domain count with an increasing alkyl chain length. Starting from *n* = 4, there are, on average, only three domains, indicating a preference for similar arrangements of alkyl chains relative to each other. For the largest systems with *n* = 10 and *n* = 12 the domain count even decreases to one, resulting in the establishment of only two distinct domains in the entire system. Notably, for *n* ≥ 4 the polar and nonpolar networks seem to coexist without destructively perturbing each other. This is a sign of stark microheterogeneity in the IL, which has also been observed in prior studies.^10,20,22,24,57−62^ At this point, it remains unclear what the exact composition of the nonpolar domains in the *n* = 2–8 systems is.

### 3.3 Cluster and Aggregation Analysis

As a next step, we aim at quantifying the size of aggregates that form within the polar and nonpolar networks. To do so, we first employ the software AGGREGATES.^31^ Within its methodology, a set of covalently bound atoms included in such an analysis is considered as an entity. Two entities belong to the same aggregate if their distance is smaller than a certain cutoff *r*_cut_, the latter usually being chosen as the first minimum of the corresponding RDF. In our specific case, the polar domains consist of 1400 entities (700 Cl^–^ and 700 [C_1_C_1_Im^+^] fragments, while each nonpolar domain owns 700 entities (the alkyl tails).

**Figure 5:**
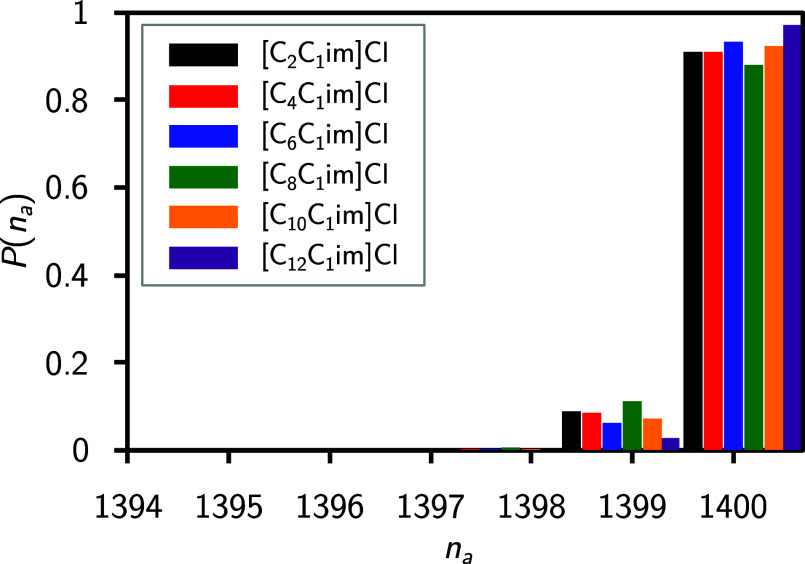
Discrete probability distribution of polar aggregate sizes *P*(*n*_*a*_), as a function of aggregate size number *n*_*a*_, for all ILs in the studied [C_*n*_C_1_Im]Cl homologous series.

**Figure 6:**
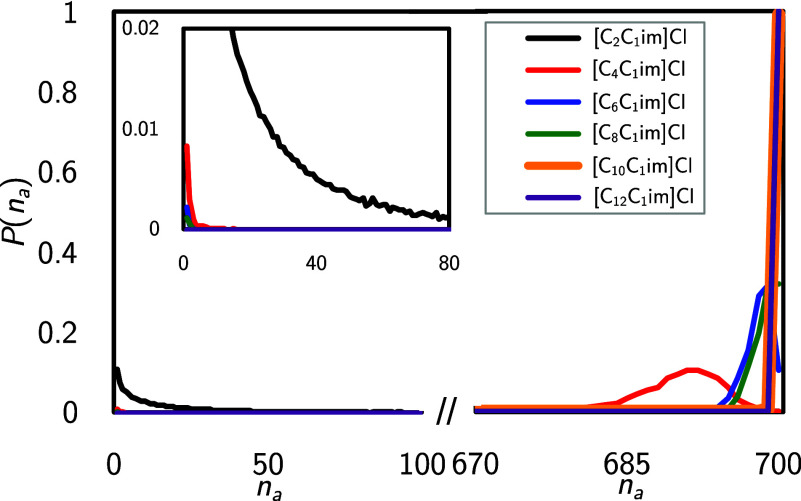
Discrete probability distribution of nonpolar aggregate sizes *P*(*n*_*a*_), as a function of aggregate size number *n*_*a*_, for all ILs in the studied [C_*n*_C_1_Im]Cl homologous series.

Figure 5 illustrates the discrete probability distribution *P* of the polar aggregates as a function of the aggregate size number *n*_*a*_. The distribution is very sharp for all studied systems, revealing that aggregate sizes of 1399 and 1400 are populated exclusively (please note that the maximum possible aggregate size is 1400). This reinforces the earlier findings from the RDF and domain analyses, demonstrating that the polar network is not only continuously distributed in the bulk but also, on average, includes ≥1399 polar entities. Moreover, it is revealed that all entities in the network (with a maximum of one exception) are within a distance of ≤530 pm to each other. Opposed to that, the aggregate size distribution of the nonpolar aggregates is more diverse, as depicted in Figure 6. It should be noted that this plot shows lines instead of bars to increase clarity. Following the trend observed during the domain analyses in section 3.2, the nonpolar network is disrupted for short alkyl chains (black line). This disruption is reflected in the most probable aggregate size being one and the distribution decaying below 1% at approximately *n*_*a*_ = 30 for the [C_2_C_1_Im]Cl system. It is worth recalling that the average nonpolar domain count was found to be 3, 1.6, and 1.3 for the *n* = 4, 6, and 8 systems, respectively. Turning to those systems (red, blue, and green lines) in Figure 6, we find that the aggregate size distribution is mostly located between 680 and 700. Thus, we conclude that a domain count > 1 cannot be attributed to multiple (more or less) evenly sized domains but, starting from the *n* = 4 system, originates from the coexistence of one large and extended domain together with a few very small domains, as can be seen in the inset at *n*_*a*_ = 1. Considering *n* = 10, 12 (yellow and violet bars, respectively), we find a probability distribution very close to one at *n*_*a*_ = 700, pointing out that the entities in the nonpolar network are truly all connected to each other within a distance of 500 pm.

The results of the aggregation analyses can be consolidated by clustering analyses from TRAVIS^30^ to address a lingering question within this work: what is the orientation of the alkyl chains relative to each other? In contrast to the methodology of AGGREGATES, where a fixed cutoff distance is used to identify clusters, TRAVIS sweeps through all possible cutoff distances and analyzes the aggregates present at every cluster size. This procedure provides additional insights that, when combined with the earlier results, contribute to a more comprehensive understanding. Particularly relevant data can, for example, be extracted from the cluster distance distribution function (CDDF) that provides information about the probability of clustering depending on the chosen cutoff distance.

**Figure 7:**
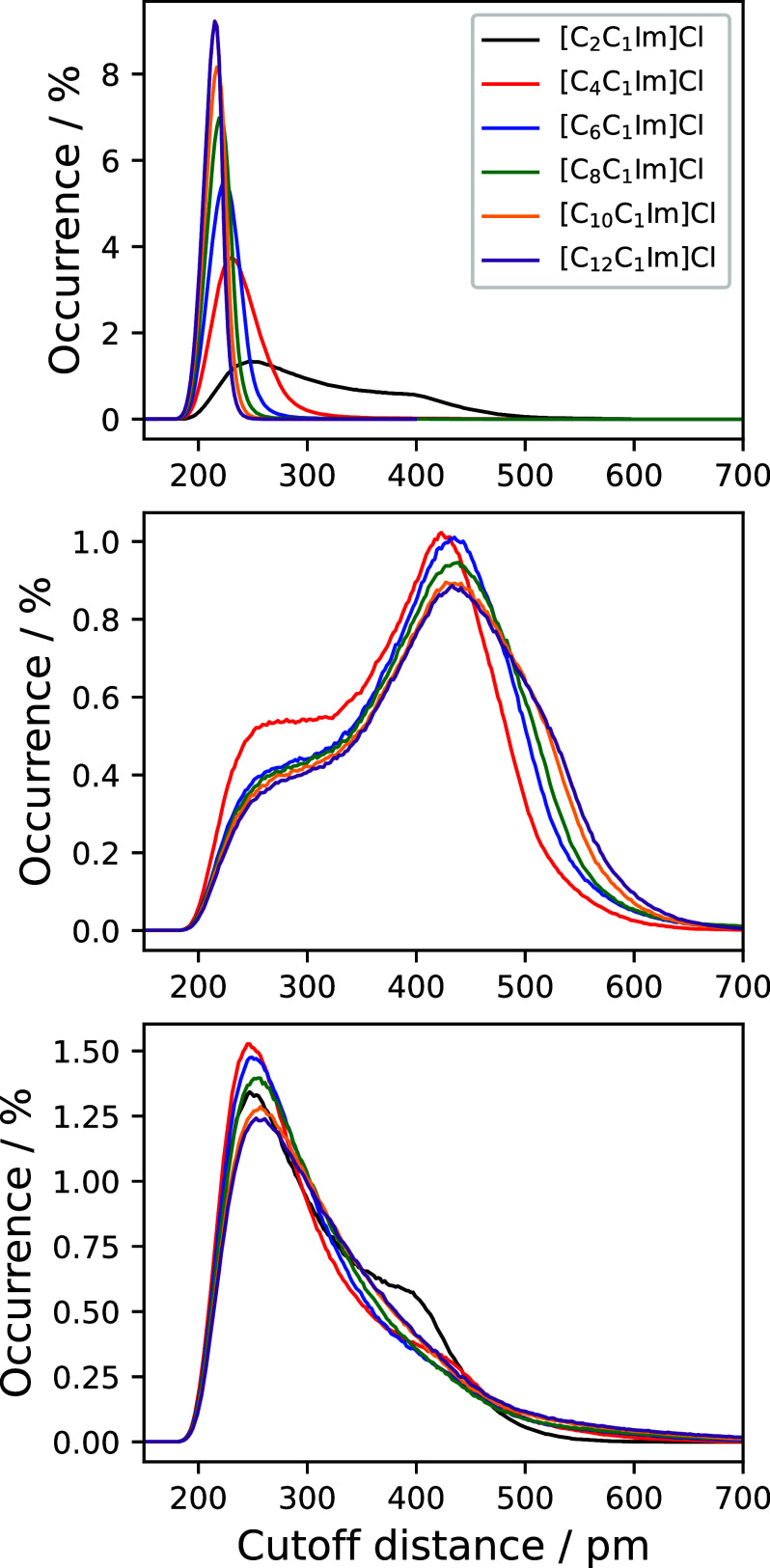
Cluster distance distribution functions of the [C_*n*_C_1_Im]Cl systems. The atoms included in the analyses are all hydrogen atoms of the alkyl chains, starting from the second carbon atom (top panel), the middle CH_2_ group (carbon and hydrogen atoms) in the alkyl chains (center panel), and the terminal CH_3_ groups (carbon and hydrogen atoms) of the alkyl chains (bottom panel). Please note that due to the shortened alkyl chain, the center panel does not show a data set for the [C_2_C_1_Im]Cl. All CDDFs were normalized to unity.

Cluster analyses of the nonpolar domains are shown in Figure 7. These feature the alkyl chains of the [C_*n*_C_1_Im^+^] ions, starting from the second carbon atom (cf. section 3.2). Three different subsets of atoms were selected: these are (top panel) all hydrogen atoms from the alkyl chains, (center panel) the central CH_2_ group of every alkyl chain, and (bottom panel) the terminal CH_3_ group of every alkyl chain. It should be noted that for [C_2_C_1_Im]Cl there is no central CH_2_ group. We note that cluster formation from entities within the same molecule is excluded in the cluster analysis, and hence, this analysis deals with *N* = 700 entities for each subset, as discussed before. The dendrograms generated from these analyses are presented in the SI. A cluster analysis on the polar domains considers the hydrogen bonding between cations and anions (i.e., the imidazolium ring protons H_A_ and the Cl^–^ anions). For brevity and clarity, the corresponding plot is available in the SI. The results of the cluster analysis align well with the findings from section 3.1, showing CDDFs that are largely independent of the alkyl chain length. A prominent peak at 260 pm is consistently observed, corresponding to the first maximum of the RDF.

The top panel shows the CDDFs of the alkyl chains when all of the hydrogen atoms (starting at the second carbon atom) are considered in the analysis. With an increasing side chain length, the broadness of the CDDFs decrease. For instance, while the [C_2_C_1_Im]Cl system displays clustering in a wide range of approximately 200 to 500 pm, the broadness decreases from 180 to 235 pm in the case of the [C_12_C_1_Im]Cl system. Interestingly, the CDDF of [C_2_C_1_Im]Cl is asymmetric and shows a bimodal course, whereas all of the other systems show one distinct and symmetric peak.

The former finding corroborates our aforementioned observations from the cation COR–COR RDF, from which we conclude that there are indeed two distinct conformations of imidazolium rings with respect to each other. The diminishing broadness of the CDDFs signifies a tendency of the alkyl chains to arrange (spatially) closer to each other with increasing chain length. This can be attributed to favorable dispersive interactions and is in accordance with the other results of the study, especially with the increasing number of contact neighbors, as displayed in Figure 4.

To gain more insight into the complex arrangement and structural features of the alkyl chains, the center and bottom panels of Figure 7 display the clustering behavior of the central CH_2_ and terminal CH_3_ groups of the chains, respectively. The CDDFs of the CH_2_ groups in the center panel feature a main peak at 450 pm and a shoulder at around 250 pm, whereas the terminal CH_3_ groups show one asymmetric peak at around 250 pm with a broad decay on the right side. From these CDDFs, it is discerned that the terminal methyl groups (bottom panel) tend to arrange in proximity to each other at a distance of around 250 pm. However, this arrangement is not universally adopted by all alkyl chains, as is evident from the broadened decay on the right side of the CDDFs. This variability is better understood when considering the CDDFs of the central CH_2_ groups in the center panel. The broader distribution of the curves in this panel indicates less well-defined arrangements compared with the terminal methyl groups. While the ends of the tails tend to cluster closely together, the conformation in the middle of the chain is less rigidly fixed.

**Figure 8:**
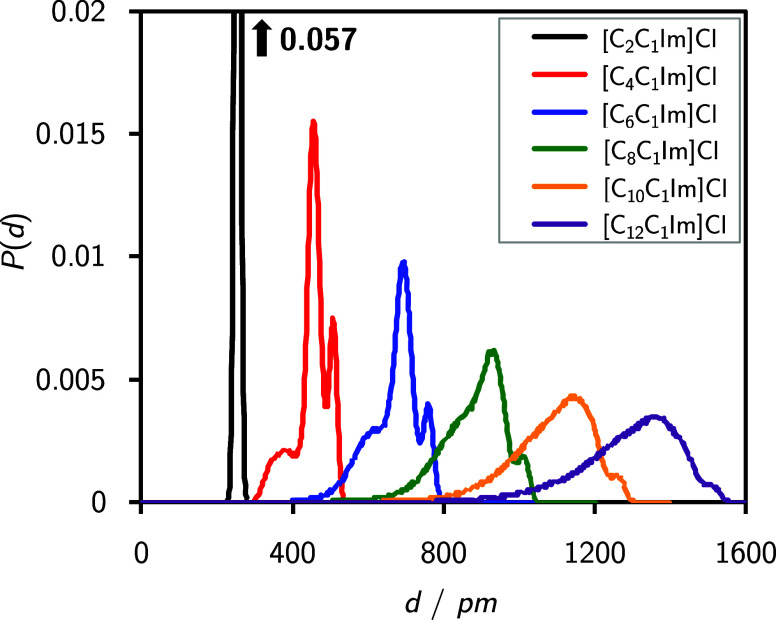
Probability distribution functions of intramolecular distances *P*(*d*), between the nitrogen atom of the cation to which the alkyl chain is attached and the terminal CH_3_ group of the alkyl chain in [C_*n*_C_1_Im]^+^ cations for all ILs in the studied [C_*n*_C_1_Im]Cl homologous series.

The varying arrangements are attributed to the conformational flexibility of the alkyl chains, capable of adopting diverse configurations ranging from linear chains to curled ball-like structures. Analysis of the alkyl chain conformation involves examining the distribution of dihedral angles along the carbon chain and assessing the intramolecular distances between the alkyl chain’s end point and the imidazolium ring. Detailed results from the dihedral distribution functions are available in the SI and indicate a pronounced increase in the probability of finding a dihedral in a 180° (linear) conformation as the alkyl chain length increases. Conversely, nonlinear arrangements become less probable. To underpin these results, we show intramolecular distances of the cations between the nitrogen atom that is next to the C_*n*_ chain and the terminal CH_3_ group in Figure 8. For *n* = 2 (black line), a single peak is observed as expected for the ethyl group. Looking at *n* = 4, 6 (red and blue lines, respectively), the distribution features two peaks and a shoulder, with the peaks being found at around 450 and 500 pm in the case of *n* = 4 as well as at 700 and 750 pm in the case of *n* = 6. Longer alkyl chains (green, yellow, and violet lines) exhibit less pronounced peaks and a slightly broader distribution, with maxima at 950, 1150, and 1400 pm for *n* = 8, 10, and 12, respectively. These findings are in qualitative agreement with a study of Margulis.^57^ With increasing chain lengths, the number of accessible conformations increases (cf. the dihedral distribution in the SI). Although the number of accessible conformations increases with longer chains, the preferred configurations are not fully linear, with some carbon chain dihedrals residing in a nonlinear state. Nonetheless, from Figure 8, it is evident that the chains do not adopt curled conformations, as indicated by the ever increasing distances at which the histogram peaks occur.

## 4 Conclusions

A systematic study has been carried out to understand the mesoscopic segregation observed in imidazolium chloride-based ionic liquids through molecular dynamics simulations. We find the polar network to be continuously distributed in the bulk, incorporating all 700 ion pairs. More specifically, we find the centers of almost all ions (with a maximum of one exception) within a short distance (corresponding to the first minimum in the respective radial distribution function) from each other. Furthermore, our analyses reveal that the structural arrangement and coordination of anions and the polar head groups of the cations do not change upon elongation of the cation alkyl chains. The aggregation of the nonpolar domain is more diverse, starting with small aggregates (disrupted for shorter alkyl chains) and growing to form aggregates that encompass all chains. The cluster analysis shows that the cluster distance distribution functions for the terminal carbon of the chain tend to arrange in proximity to each other, as seen in one asymmetric peak, while the CDDFs for the central CH_2_ groups present a wider distribution, indicating that the conformation in the middle of the chain is less rigidly fixed. From our analyses, we conclude that the cation tails preferably arrange in line, allowing them to maximize favorable noncovalent interactions. In conclusion, we highlighted how the two software programs TRAVIS and AGGREGATES complement each other to unravel complex structural arrangements in bulk systems with the example of the common IL [C_2_C_1_Im][Cl]. Specifically, the aggregate analysis can provide information about the size of aggregates, while the cluster analysis can offer insights into the morphology within these aggregates. Consequently, one can utilize cluster analysis to determine distances for use in aggregate analysis, or alternatively, once the size of the aggregates is known, cluster analysis can be employed to gain information about their morphology.

## Supporting Information Available

Additional radial distribution functions and temporal RDF convergence, additional cluster analyses, and dihedral distribution functions of the cation alykl chains (PDF)
