# Supplementary material for: Unraveling the Morphology of [CnC1Im]Cl Ionic Liquids Combining Cluster and Aggregation Analyses
Source: J Phys Chem B. 2024 Apr 15;128(16):3937–45. doi: 10.1021/acs.jpcb.3c08317 (PMC11056978; doi:10.1021/acs.jpcb.3c08317)
Supplement: Supplementary file 1 — jp3c08317_si_001.pdf [file jp3c08317_si_001.pdf]

Supporting Information: Unraveling the Morphology of [C_nC_1Im]Cl Ionic Liquids Combining Cluster and Aggregation Analyses

Tom Frömbgen,[†,‡] José Nuno Canongia Lopes,[¶] Barbara Kirchner,[†] and Karina Shimizu[*,¶]

†*Mulliken Center for Theoretical Chemistry, University of Bonn, Beringstraße 4-6, D-53115 Bonn, Germany*

‡*Max-Planck-Institut für Chemische Energiekonversion, Stiftstrasse 34-36, D-45470 Mülheim an der Ruhr, Germany*

¶*Centro de Química Estrutural, Institute of Molecular Sciences, Instituto Superior Técnico, Universidade de Lisboa, Av Rovisco Pais 1, 1049 001 Lisboa, Portugal*

E-mail: karina.shimizu@tecnico.ulisboa.pt

1 Additional Radial Distribution Functions

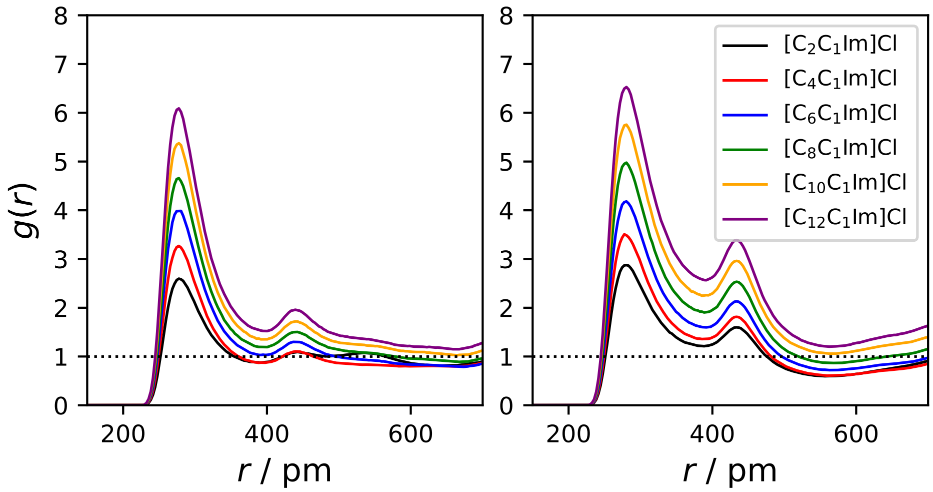

Figure S1: Radial distribution functions between the chloride anions and the aliphatic CH_2 (left panel) and CH_3 groups (right panel) that are closest to the imidazolium ring for all systems studied.

In fig. S1, we show $H{-}Cl^-$ RDFs, where the H atoms denote the aliphatic CH_2 and CH_3 groups directly attached to the imidazolium ring. These interactions are important for hydrogen bonding as well, as for example outlined in Ref. S1.

Tackling the question of the convergence behavior of the static properties discussed in our manuscript, in fig. S2 we show the time evolution of the RDFs between Cl^- and the center of mass (COM) of the cations for the $[C_2C_1Im]Cl$ (left) and $[C_{12}C_1Im]Cl$ systems (right). These RDFs correspond to the black and violet data sets in the center panel of Figure 2 of the main text. As evident, the RDFs are converged within 2 ps. Note that the overall length of our trajectories is 10 ps. We further note that other properties discussed in our manuscript behave similarly with respect to their convergence.

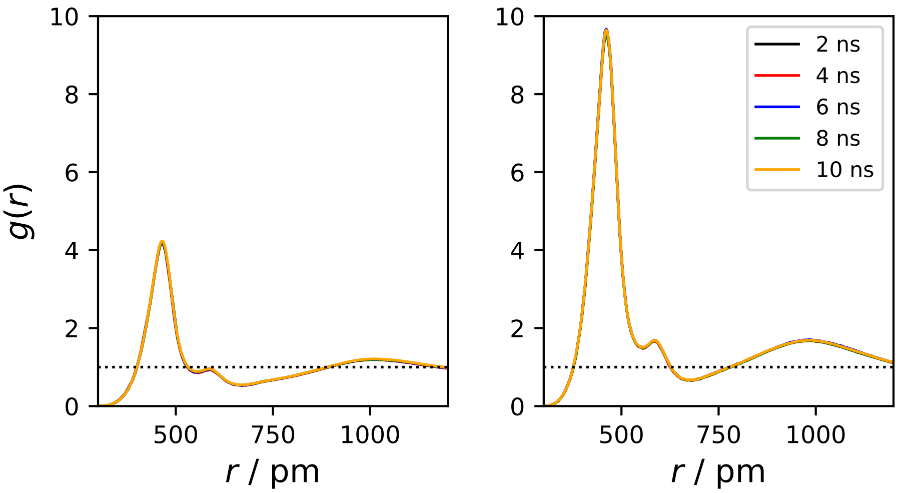

Figure S2: Time-dependent radial distribution functions between Cl^- and the center of mass (COM) of the cations for the $[C_2C_1Im]Cl$ (left) and $[C_{12}C_1Im]Cl$ systems (right).

2 Additional Cluster Analyses

2.1 Hydrogen bonding between the ions

Figure S3 shows the CDDFs and CSDFs obtained from a cluster analysis between the cations' $H_{2,4,5}$ and the Cl^- anions. The CDDFs of all systems show a symmetric shape of the CDDF with a maximum at $r_{cut} = 260\,pm$ which is identical to the maximum values of the corresponding RDFs discussed in the manuscript. These results support the suggestion that there are no structural changes in the interaction modes of H_A and Cl^- with altering the imidazolium alkyl chain length.

2.2 Interactions in the nonpolar domains

To further investigate the clustering behavior in the non-polar domain, the corresponding cluster polymer distribution functions (CPDFs) from the $[C_2C_1Im]Cl$ and $[C_{12}C_1Im]Cl$ systems are presented in the top and bottom panel of fig. S4, respectively. In the CPDF plots, the gray surface corresponds to the monomers (1-mers) and the red surface to the N-mer (700-mer). The starting point of the decay of population of the monomers corresponds to the initial rise of the respective CDDF. Accordingly, the point at which the N-mer population

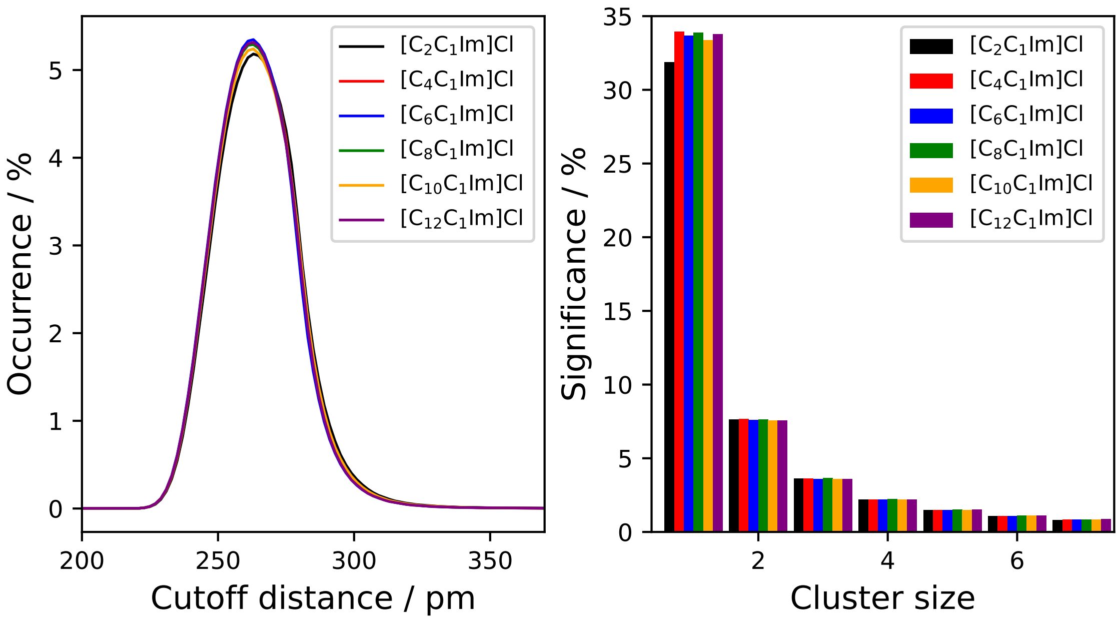

Figure S3: Cluster distance distribution functions (left) and cluster significance distribution functions (right) featuring the hydrogen bonding between cations and anions. The atoms included in this analysis comprise the imidazolium ring protons H_A and the Cl^- anions. We note that clusters were only allowed to be formed via contacts of hydrogen with chloride. Contacts of one entity with another entity of the same type were excluded from the analysis.

reaches 100 % is equivalent to the point where the CDDF decays to zero. It should be noted that cumulative probability of all cluster sizes is always 100 % because every particle has to be part of one cluster. Figure S4 visualizes how the average distance between the alkyl chains decreases with increasing alkyl chain length. This gives rise to the suggestion that the non-polar network is disrupted in systems with short alkyl chains.

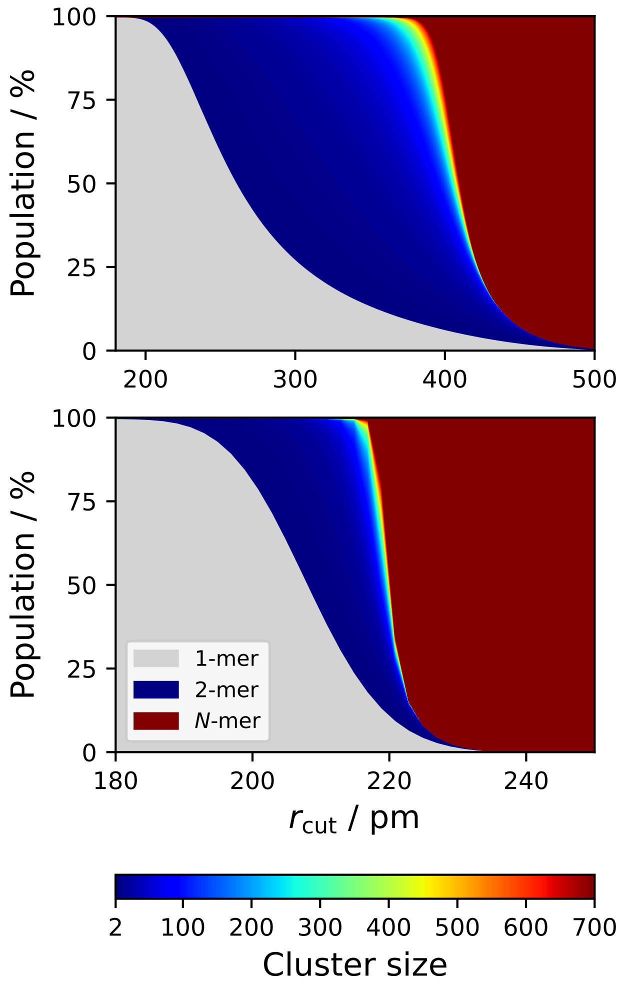

Figure S4: Cluster polymer distribution functions from the [C_2C_1Im]Cl (top) and [$C_{12}C_1$Im]Cl (bottom) systems.

2.3 Dendrograms of alkyl chains

For reasons of completeness, the dendrograms corresponding to the top, center and bottom panel from fig. 6 of the manuscript are presented in figs. S5 to S7, respectively.

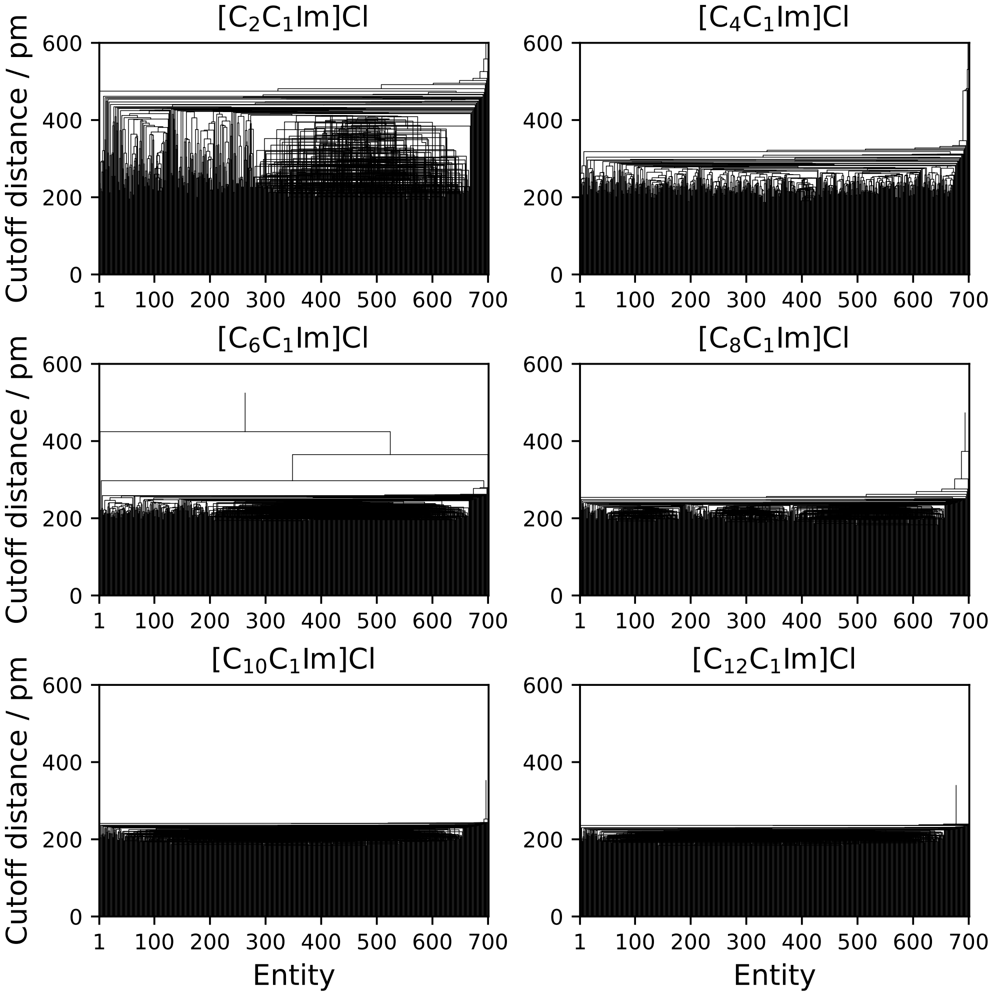

Figure S5: Dendrograms of the [C_nC_1Im]Cl ionic liquids, considering all hydrogen atoms of the alkyl chains starting from the second carbon atom of the side chain.

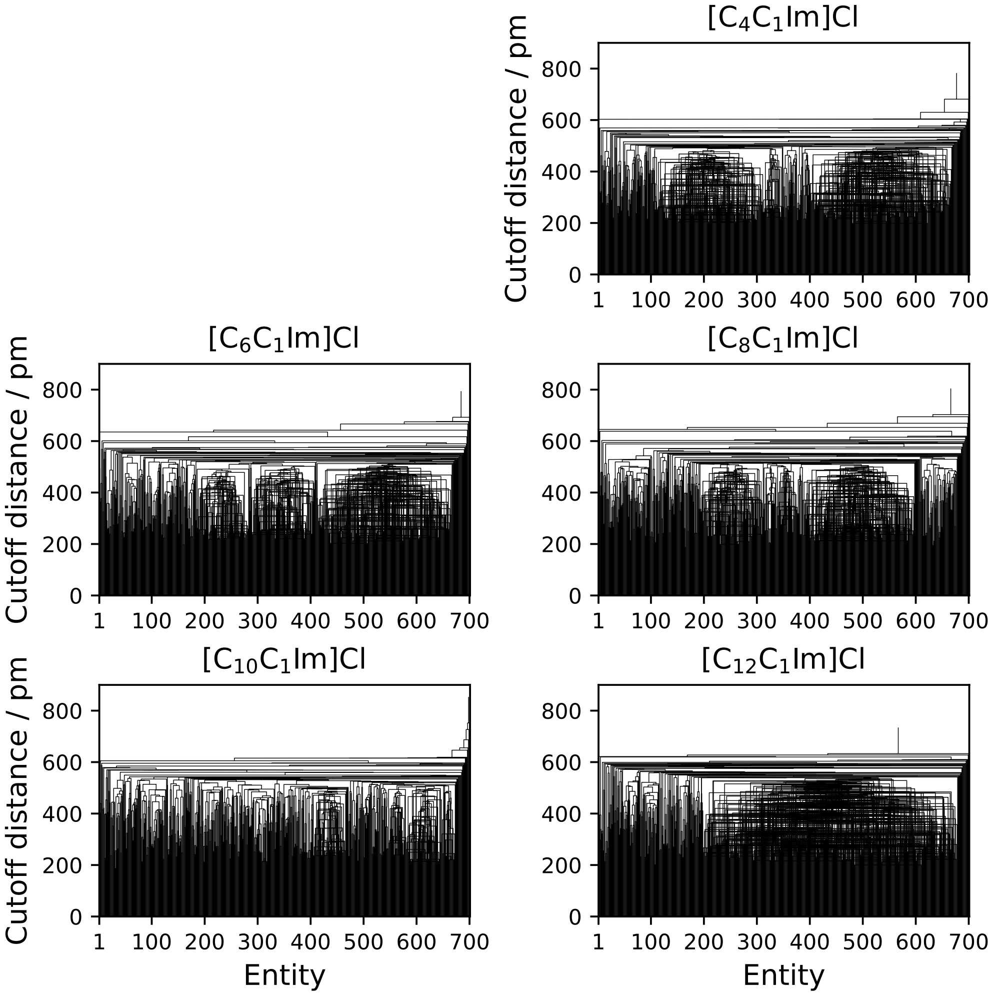

Figure S6: Dendrograms of the $[C_nC_1Im]Cl$ ionic liquids, considering the carbon and hydrogen atoms of the center CH_2 group of the alkyl chains.

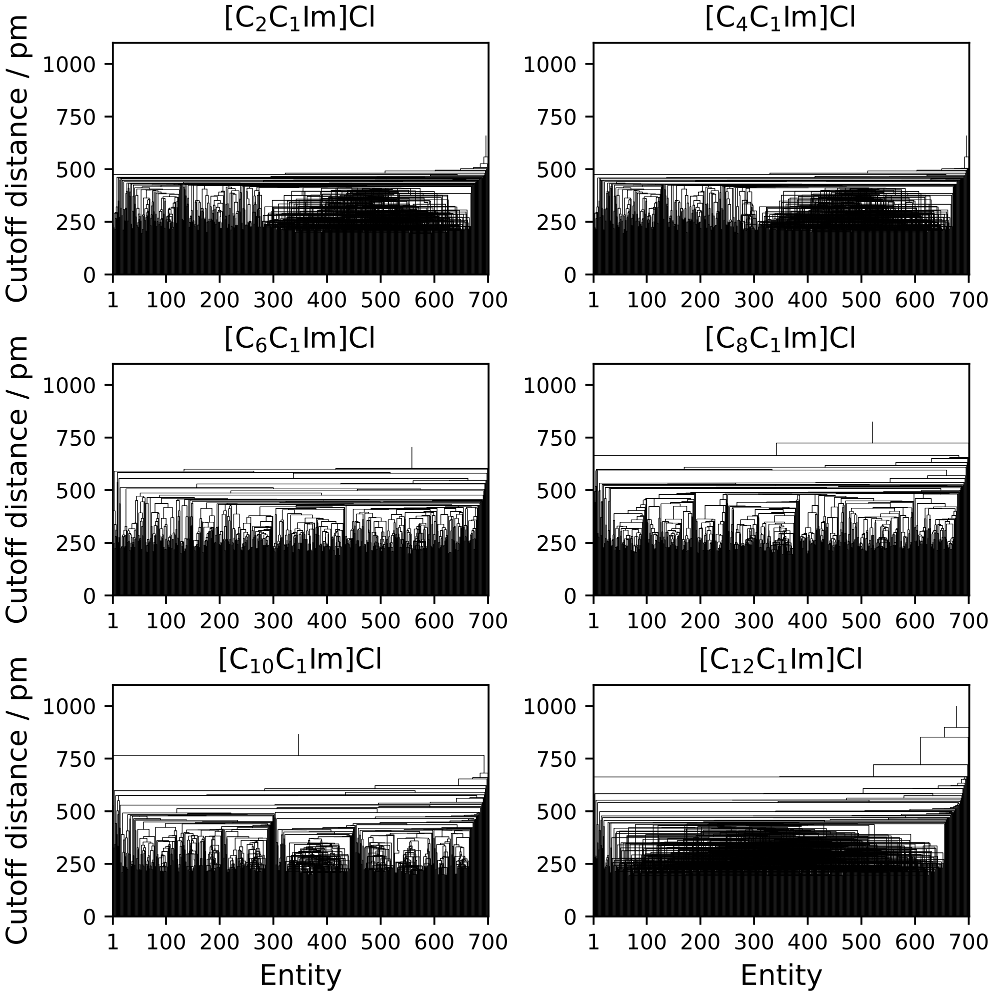

Figure S7: Dendrograms of the $[C_nC_1Im]$cl ionic liquids, considering the carbon and hydrogen atoms of the terminal CH_2 group of the alkyl chains.

3 Dihedral distribution of the cation alkyl chains

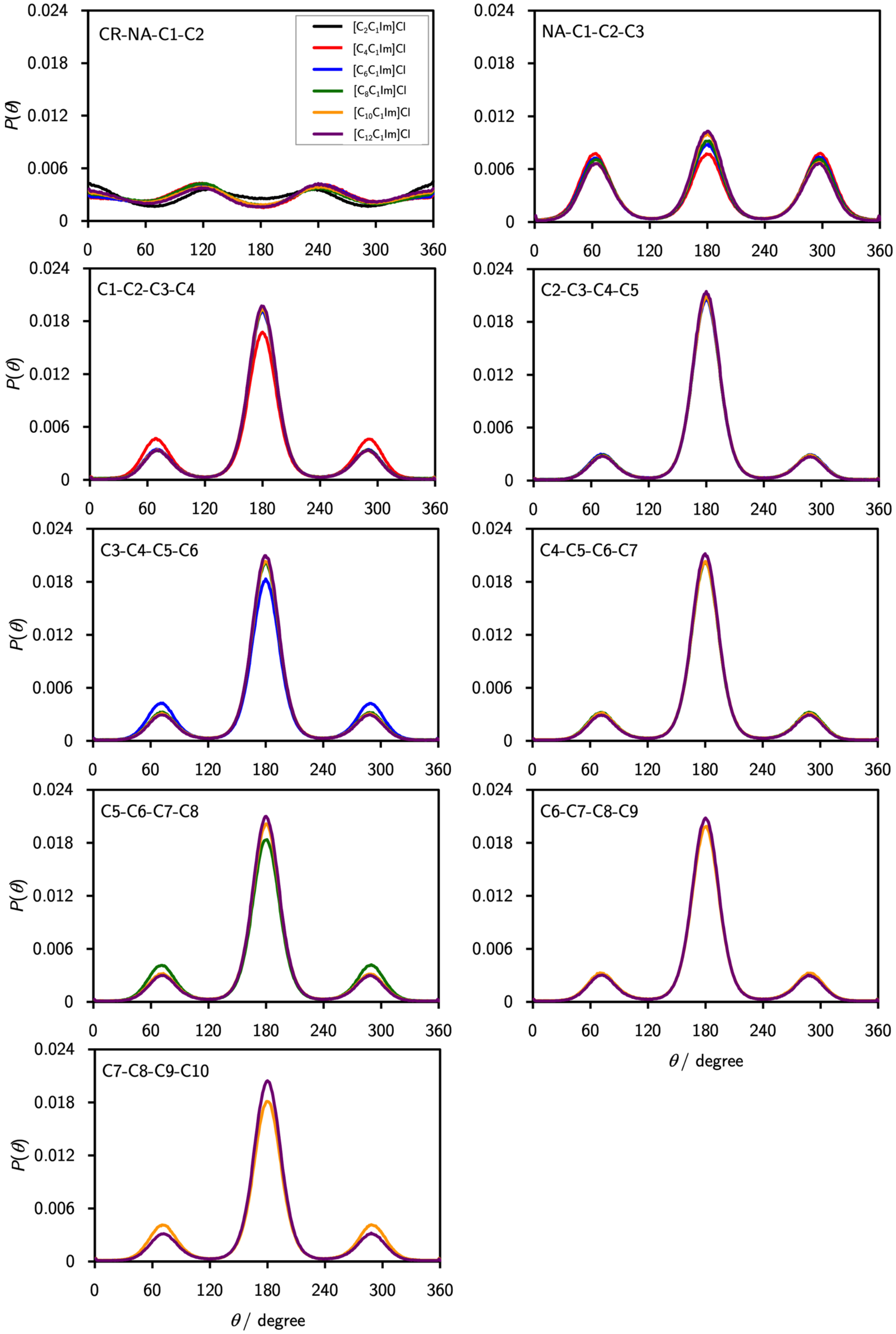

Figure S8: Probability distribution functions $P(\theta)$ of the dihedral angles θ of the alkyl tails in $[C_nC_1Im]Cl$ ionic liquids. Each panel shows the distribution of one distinct dihedral as a comparison between the different types of cations that possess this dihedral.

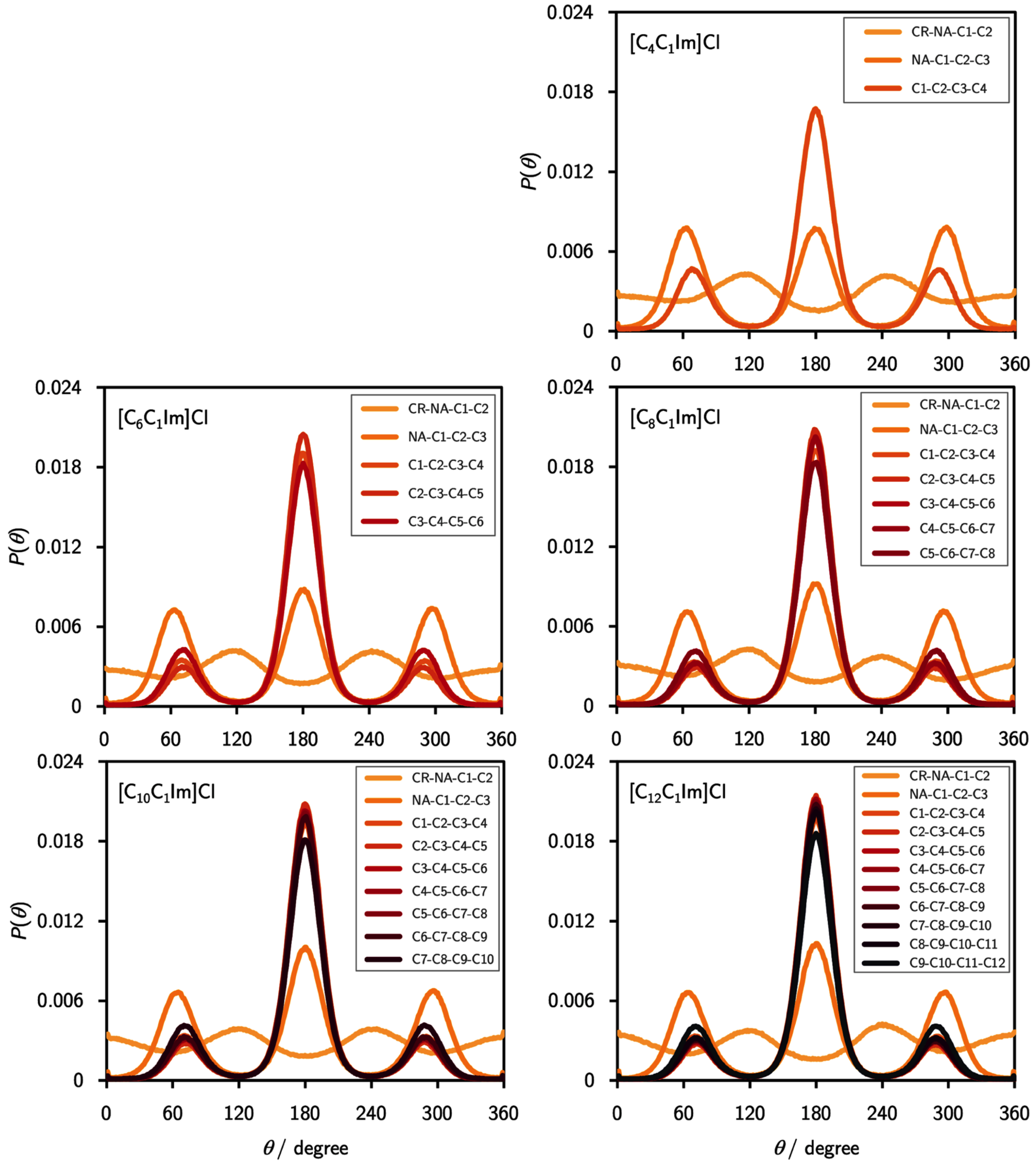

Figure S9: Probability distribution functions $P(\theta)$ of the dihedral angles θ of the alkyl tails in $[C_nC_1Im]Cl$ ionic liquids. Each panel shows features one type of cation, and shows the distribution of all dihedrals present in this type of cation.

References

(S1) Skarmoutsos, I.; Dellis, D.; Matthews, R. P.; Welton, T.; Hunt, P. A. Hydrogen Bonding in 1-Butyl- and 1-Ethyl-3-methylimidazolium Chloride Ionic Liquids. *116*, 4921–4933.
